# Evaluation of the safety and efficacy of XAV-19 in patients with COVID-19-induced moderate pneumonia: study protocol for a randomized, double-blinded, placebo-controlled phase 2 (2a and 2b) trial

**DOI:** 10.1186/s13063-021-05132-9

**Published:** 2021-03-09

**Authors:** Benjamin Gaborit, Bernard Vanhove, Marie-Anne Vibet, Aurélie Le Thuaut, Karine Lacombe, Vincent Dubee, Florence Ader, Virginie Ferre, Eric Vicaut, Jéremie Orain, Morgane Le Bras, Anne Omnes, Laetitia Berly, Alexandra Jobert, Pascale Morineau-Le Houssine, Karine Botturi, Régis Josien, Laurent Flet, Nicolas Degauque, Sophie Brouard, Odile Duvaux, Alexandra Poinas, François Raffi, POLYCOR study group, Eric Dailly, Thomas Guimard, Cécile Braudeau, Denis Malvy, Jean-François Faucher, Gabriela Illes Hajnal, Marc-Olivier Vareil, Mariam Roncato-Saberam, Laurent Vacher, Charlotte Biron, Maeva Lefebvre, Géraldine Gallot, Paul Le Turnier, Colin Deschanvres, Raphael Lecomte, Marie Chauveau, Anne-sophie Lecompte, Matthieu Grégoire, Ronan Bellouard, Guillaume Deslandes, Zineb Ouazene, Diane Bollens, Thibault Chiarabani, Jessica Krause-Le Garrec, Agathe Becker, Pierre Chauvelot, Anne Conrad, Tristan Ferry, Patrick Miailhes, Cécile Pouderoux, Sandrine Roux, Claire Triffault-Fillit

**Affiliations:** 1 CHU Nantes, Department of Infectious Disease, Clinical Investigation, Nantes, France; 2 CHU Nantes and Inserm, Clinical Investigation Centre CIC1413, Nantes, France; 3 Xenothera, Nantes, France; 4 CHU Nantes, Sponsor Department, Nantes, France; 5 Institut Pierre Louis d’Epidémiologie et de Santé Publique, Sorbonne Université, INSERM, AP-HP, Hôpital Saint-Antoine, Service des Maladies Infectieuses et Tropicales, Paris, France; 6 CHU Angers, Service de Maladies Infectieuses et Tropicales, Angers, France; 7 Centre International de Recherche en Infectiologie (CIRI), Inserm 1111, Université Claude Bernard Lyon 1, CNRS, UMR5308, Ecole Normale Supérieure de Lyon, Université Lyon, F-69007 Lyon, France; 8 Département des Maladies infectieuses et tropicales, Hospices Civils de Lyon, F-69004 Lyon, France; 9 CHU Nantes, Virology Laboratory, Nantes, France; 10 APHP, Department of Biostatistics, Université Paris-Diderot, Sorbonne-Paris Cité, Fernand Widal Hospital, Paris, France; 11 CHU Nantes, Partnership and Innovation Department, Nantes, France; 12 Nantes Université, CHU Nantes, Inserm, Centre de Recherche en Transplantation et Immunologie, UMR 1064, ITUN, F-44000 Nantes, France; 13 CHU Nantes, Laboratoire d’Immunologie, Nantes, France; 14 CHU Nantes, Pharmacy Department, Nantes, France; 15 CHU Nantes, Nantes Université, Institut de Transplantation Urologie Néphrologie (ITUN), Nantes, France

**Keywords:** COVID-19, Anti-SARS-CoV-2 antibodies, Moderate pneumonia, Immunotherapy, Randomized controlled trial, Phase 2

## Abstract

**Background:**

Early inhibition of entry and replication of the severe acute respiratory syndrome coronavirus 2 (SARS-CoV-2) is a very promising therapeutic approach. Polyclonal neutralizing antibodies offers many advantages such as providing immediate immunity, consequently blunting an early pro-inflammatory pathogenic endogenous antibody response and lack of drug-drug interactions.

By providing immediate immunity and inhibiting entry into cells, neutralizing antibody treatment is of interest for patient with COVID-19-induced moderate pneumonia. Convalescent plasma to treat infected patients is therefore a relevant therapeutic option currently under assessment (CORIMUNO-PLASM NCT04324047). However, the difficulties of collecting plasma on the long term are not adapted to a broad use across all populations.

New polyclonal humanized anti-SARS-CoV2 antibodies (XAV-19) developed by Xenothera and administered intravenous. XAV-19 is a heterologous swine glyco-humanized polyclonal antibody (GH-pAb) raised against the spike protein of SARS-CoV-2, blocking infection of ACE-2-positive human cells with SARS-CoV-2.

**Methods:**

Pharmacokinetic and pharmacodynamic studies have been performed in preclinical models including primates. A first human study with another fully representative GH-pAb from Xenothera is ongoing in recipients of a kidney graft. These studies indicated that 5 consecutive administrations of GH-pAbs can be safely performed in humans.

The objectives of this 2-step phase 2 randomized double-blinded, placebo-controlled study are to define the safety and the optimal XAV-19 dose to administrate to patients with SARS-CoV-2 induced moderate pneumonia, and to assess the clinical benefits of a selected dose of XAV-19 in this population.

**Discussion:**

This study will determine the clinical benefits of XAV-19 when administered to patients with SARS-CoV-2-induced moderate pneumonia. As a prerequisite, a first step of the study will define the safety and the dose of XAV-19 to be used.

Such treatment might become a new therapeutic option to provide an effective treatment for COVID-19 patients (possibly in combination with anti-viral and immunotherapies). Further studies could later evaluate such passive immunotherapy as a potential post-exposure prophylaxis.

**Trial registration:**

ClinicalTrials.gov NCT04453384, registered on 1 July 2020, and EUDRACT 2020-002574-27, registered 6 June 2020.

**Supplementary Information:**

The online version contains supplementary material available at 10.1186/s13063-021-05132-9.

## Administrative information

The order of the items has been modified to group similar items (see http://www.equator-network.org/reporting-guidelines/spirit-2013-statement-defining-standard-protocol-items-for-clinical-trials/).

**Table:** 

**Title {1}**	A randomized, double-blinded, placebo-controlled phase 2 (2a and 2b) study to evaluate the safety and efficacy of XAV-19 in patients with COVID-19 induced moderate pneumonia
**Trial registration {2a and 2b}**	Registration number NCT04453384, first published on July 1, 2020. https://clinicaltrials.gov/ct2/show/NCT04453384 and EudraCT number 2020-002574-27, first published on June 15, 2020. https://www.clinicaltrialsregister.eu/ctr-search/trial/2020-002574-27/FR
**Protocol version {3}**	The updated protocol is at version 1.4 on August 4, 2020.
**Funding {4}**	This study is supported by Public Investment Bank, also known as BPI France in the framework of the “Investment for the Future” programme (Programme d’Investissements d’Avenir).
**Author detail {5a}**	Benjamin Gaborit, Pascale Morineau, Jeremie Orain, Morgane Le Bras and François Raffi belong to the CHU Nantes, Department of Infectious Disease and the Clinical Investigation Centre CIC1413 (INSERM and CHU Nantes). Alexandra Poinas belongs exclusively to the Clinical Investigation Centre CIC1413 (INSERM and CHU Nantes). Marie-Anne Vibet; Aurélie Le Thuaut, Laetitia Berly, Anne Omnes and Alexandra Jobert belong to the CHU Nantes, Sponsor Department. The other authors belonging to the Nantes University Hospital are Karine Botturi (Partnership and Innovation Department), Regis Josien (Immunology laboratory), Sophie Brouard and Nicolas Degauque (Center of Research in Transplantation and Immunology) Virginie Ferré (Virology Laboratory) and Laurent Flet (Pharmacy department). Odile Duvaux and Bernard Vanhove belong to the Xenothera Laboratory. Florence Ader, Karine Lacombe and Vincent Dubee are principal investigators, belong respectively to the Lyon University Hospital (Hospices Civils de Lyon), AP-HP (Saint-Antoine Hospital) and Angers University Hospital. Eric Vicaut belongs to AP-HP, Fernand Widal Hospital.
**Name and contact information for the trial Sponsor {5b}**	Karine Botturi and Laetitia Berly are the sponsor project managers and they manage the logistics of the trial. Any request for POLYCOR information can be made via this e-mail address: BP-direction-de-la-recherche@chu-nantes.fr.
**Role of sponsor {5c}**	All the submissions/declarations were made by the Sponsor Department at CHU Nantes, which of course manages the quality of the data collected. The data collected during the study will be processed electronically in accordance with the requirements of the CNIL, the French Data Protection Authority and with the European and French regulations regarding the safety concerns. Requests for substantial modifications of the protocol should be addressed by the sponsor for approval or notification to French regulatory authorities and/or the Ethical Review Board concerned in compliance with Law 2004-806 of 9 August, 2004 and its implementing decrees.

## Introduction

### Background and rationale{6a}

In December 2019, coronavirus disease 2019 (COVID-19) emerged in Wuhan, China. After10 months, COVID-19 causes more than 35,216,168 confirmed cases and more than 1,037,557 has been announced (https://coronavirus.jhu.edu/map.html viewed on 5 October 2020).

In the lack of effective therapy and without any control of the virus’ spread, the World Health Organization declared a public health emergency of international concern on 30 January 2020.

Severe acute respiratory syndrome coronavirus 2 (SARS-CoV-2) is a protein-enveloped RNA virus [1] that induces influenza-like symptoms (fever, cough, dyspnea, vomiting, diarrhea) requiring admission to hospital in 20% of cases for respiratory illnesses and to intensive care unit in 5% of cases for severe diseases [2]. Over 25% of patients develop acute respiratory distress during the second week of disease with the onset of severe acute respiratory distress syndrome (ARDS) [3].

SARS-CoV-2 infection has been reported to induce both direct organ damage [4, 5] and inappropriate immune response causing “viral sepsis” [6–8]. The pathophysiological mechanisms of severe infection are poorly understood [9]. In the most critical cases, the overwhelming immunological reactions induced by systemic multiorgan viral invasion lead to multiorgan failure and subsequent mortality [10]. It is likely that both antivirals and blockage of inflammatory pathways are needed to optimize responses.

Due to the urgent need to develop available therapies to manage the pulmonary complications of COVID-19, many efforts continue to develop antivirals and immunotherapies against COVID-19.

By providing immediate immunity and inhibiting entry into cells, convalescent plasma has been demonstrated to improve survival rate of patients with SARS-CoV infections in 2003 [11]. SARS-CoV-2 has been shown to use the same cell entry receptor as SARS-CoV, angiotensin-converting enzyme 2 (ACE2) [12, 13]. With the inhibition of SARS-CoV-2 spike glycoprotein (S)-mediated entry into cells, convalescent plasma is a promising approach to treat patients infected with SARS-CoV-2. Early administration of neutralizing Abs may inhibit viral entry and replication and consequently blunt an early pro-inflammatory response.

Xenothera, Nantes-based biotech company spin-off from the University of Nantes, has developed a novel polyclonal glyco-humanized (GH-pAb) anti-SARS-CoV2 swine antibody named “XAV-19”. GH-pAbs described by Vanhove et al. and Salama et al. [14, 15] have been evaluated for safety, pharmacokinetics, and pharmacodynamics effects in preclinical assessments in non-human primates and in volunteer kidney recipient patients dosed daily for 5 days at the five doses levels of 0.6 mg/kg, 1 mg/kg, 3 mg/kg, 6 mg/kg, and 8 mg/kg [16]. Forty milligrams per kilogram is therefore the maximal cumulative dose that has been administered in human. While the study is still ongoing, the Data and Safety Monitoring Committee (DSMC) agreed to pursue to the second segment of the study (therapeutic dose) where the GH-pAb will be administered 5 times at 8 mg/kg (cumulative dose of 40 mg/kg).

#### Efficacy of neutralizing antibodies (Abs)

In mice, neutralizing Ab against SARS-CoV elicited by primary infection can protect from secondary-infection and prevent SARS-CoV replication in respiratory tract of naïve mice [11]. Small retrospective case-comparisons studies in patients with SARS-CoV and SARS-CoV-2 suggested a case fatality rate reduction after convalescent plasma treatment [17–19]. An early administration at a time where pathology may be driven mainly by viral replication appears most suitable.

#### Safety profile

The issue of the potential toxicity associated with convalescent plasma and the difficulty in collecting it is a major limitation to the widespread use of this treatment. XAV-19 is a novel GH-pAb from Xenothera Laboratory technology. Per se, XAV-19 has never been administered in human. However, it is chemically comparable to LIS1, a lymphodepleting GH-pAb introduced in the clinic by Xenothera in 2019. LIS1 has been safely administered to kidney graft recipients at cumulated doses up to 40 mg/kg, representing 40-fold the lowest dose foreseen for XAV-19 in the POLYCOR study.

#### New polyclonal glyco-humanized anti-SARS-CoV2 antibodies formulation

The XAV-19 drug substance (DS) is a liquid, colorless, sterile solution concentrated at 5.0 ± 0.5 mg/mL. XAV-19 is generated as follows: the active substance manufacturing process starts from a pool of swine serum collected after immunization with a recombinant antigen (SARS-Cov-2 spike protein domain). The IgG fraction is being purified with a downstream process similar to processes used for mAb purification.

#### POLYCOR study

Knowing GH-pAbs are well tolerated in human and that neutralizing Abs can be an effective therapeutic approach during SARS disease, the POLYCOR aims to address, in randomized double-blinded, placebo-controlled study the efficacy of polyclonal humanized anti-SARS-CoV2 antibodies (XAV-19) in patients with COVID-19-induced moderate pneumonia.

POLYCOR is divided into 2 steps: the phase 2a will be a double ascending dose (DAD), double-blinded, placebo-controlled randomized study to select the optimal dose of XAV-19 for the phase 2b. The phase 2b (double fixed dose (DFD)) will be a double-blinded, placebo-controlled randomized study to assess clinical benefit and safety of the phase 2a selected dose of XAV-19 in 352 hospitalized adults with COVID-19-associated moderate pneumonia.

### Objectives {7}

#### The objectives of phase 2a study

The primary objectives are to (1) evaluate the titers of XAV-19 in treated patients versus placebo-treated patients at day 8 and (2) assess the safety and tolerability of XAV-19.

The secondary objectives of the study are to (1) characterize pharmacokinetics (PK) of XAV-19 infected patients over the time from D1 to D29, (2) evaluate the antibody titers of XAV-19 and to compare group 1 treated patients versus group 2 treated patients at day 8, and (3) describe groups of patients according to clinical variables (duration of supplemental oxygen, transfer in intensive care unit (ICU), normalization of fever, titer of biomarkers, ordinal scale assessed at day 15, hospital length of stay).

Exploratory objectives will be to assess the effects of neutralizing antibodies use on virus-induced immune response on longitudinal follow-up and to identify targets for “immuno-monitoring” for the next phase of the study and to investigate the immunogenicity of COVID-19 during treatment with XAV-19.

#### The objectives of phase 2b study

The primary objective of the study is to compare duration until weaning supplemental oxygen in the two groups of treatment before or at day 15.

The secondary objectives of the study are the comparison between two groups of:

1. Evolution of National Early Warning Score (NEWS 2) [20]
2. Changes in the 7-point ordinal scale between baseline and day 15
3. Improvement of one category from admission using the 7-point ordinal scale
4. Normalization of fever
5. Duration of oxygen therapy over 29 days
6. Oxygen requirement over 29 days
7. Time to weaning in supplemental oxygen and proportion without O2 requirement at D15
8. Transfer to ICU with need for invasive mechanical ventilation or high flow O2
9. Hospital length of stay
10. All cause of mortality
11. Safety of XAV-19

To note*,* two ancillary studies will complete the phase 2b. For the pharmacokinetic study, 16 patients will be included, as for the immuno-monitoring study 30 patients will be included. Analyses performed will be identical to those performed in the phase 2a study.

Study sites involved in these ancillary studies will propose to their first patients to participate to these studies. Patients can participate simultaneously to both studies.

The objective of the pharmacokinetic study is to characterize pharmacokinetics (PK) of XAV-19-infected patients over time from D1 to D29. Pharmacokinetic analysis corresponds to antibody titer measurements at day 1 (pre-dose, post-dose), day 3, day 5 (pre-dose, post-dose), day 8, day 15, and day 29.

Objectives will be to assess the effects of neutralizing antibodies use on virus-induced immune response on longitudinal follow-up and to identify targets for “immuno-monitoring” for the next phase of the study and to investigate the immunogenicity of COVID-19 during treatment with XAV-19.

### Trial design {8}

As already mentioned, POLYCOR is a phase 2 study with the aim to define the optimal XAV-19 dose to administrate to moderate COVID-19 patients and to evaluate its safety and efficacy. POLYCOR is divided in 2 steps. The phase 2a will be a double ascending dose (DAD), double-blinded, placebo-controlled randomized study to select the optimal dose of XAV-19 for the phase 2b. The phase 2b (double fixed dose, (DFD)) will be a double-blinded, placebo-controlled randomized study to assess clinical benefit and safety of the phase 2a selected dose of XAV-19 in 352 hospitalized adults with COVID-19-associated moderate pneumonia.

## Methods: participants, interventions and outcomes

### Study setting {9}

This study is multi-centered and national. The sixteen patients of phase 2a trial will be recruited in 4 hospitals, and more precisely in the Infectious Diseases Departments of University Hospital of Nantes, Lyon, Angers, and the Hospital of Saint-Antoine (Paris).

As soon as phase 2a is completed, phase 2b will begin with 31 hospital centers in addition to the 4 in phase 2a. These 35 centers will cover the entire national French territory.

### Eligibility criteria {10}

The population to be studied is adults (male or female), over 18 years old, hospitalized for COVID-19 infection confirmed by a positive RT-PCR of less than 10 days’ duration, associated with pneumonia and Sp02 ≥ 94% on oxygenotherapy. The patients will be recruited in the investigational sites declared on this study.

Women of childbearing potential (WOCBP) must use appropriate method(s) of contraception during the clinical trial (oral contraception, implant or intrauterine device (IUD). Pregnant women, women of childbearing potential (WOCBP) without effective contraception, women who are nursing, or patient under guardianship or trusteeship will not be included in the study. Furthermore, all sexually active male subjects must agree to use an adequate method of contraception throughout the study period and for 90 days after the last dose of study drug and agree to no sperm donation until the end of the study, or for 90 days after the last dose of XAV-19, whichever is longer.

The subjects cannot participate simultaneously in other interventional studies and until 3 months after the end of their participation to this study. All the inclusion and exclusion criteria are reported in Table 1.

**Table 1:** Inclusion and exclusion criteria

Inclusion criteria	Exclusion criteria
❖ Male or female ≥18 years and ≤ 85 years	❖ Evidence of multiorgan failure (severe COVID-19)
❖ Women of childbearing potential (WOCBP) must use appropriate method(s) of contraception during the clinical trial (oral contraception, implant or IUD	❖ Mechanically ventilated (including ECMO)
❖ Willing and able to provide written informed consent prior to performing study procedures	❖ Receipt of immunoglobulins or any blood products in the past 30 days
❖ Hospitalized for COVID-19	❖ Psychiatric or cognitive illness or recreational drug/alcohol use that in the opinion of the investigator, would affect subject safety and/or compliance
❖ Positive SARS-CoV-2 RT-PCR in any body specimen (nasopharynx, saliva, sputum) ≤ 10 days before enrolment	❖ End-stage renal disease (eGFR < 15 ml/min/1,73 m^2^)
❖ Evidence of pulmonary involvement (on lung examination [rales/crackles] and/or chest-imaging [Chest X-ray or computed tomography])	❖ Child-Pugh C stage liver cirrhosis
❖ Requiring O2 supplement ≤6 L/min at screening	❖ Decompensated cardiac insufficiency
❖ Requiring O2 supplementation with SpO2 ≥ 94% on O2 therapy at screening	❖ **History of active drug abuse**
❖ First onset of COVID-19 symptoms ≤10 days, among fever and/or chills, headache, myalgias, cough, shortness of breath, whichever as occurred fist	❖ Known allergy, hypersensitivity, or intolerance to the study drug, or to any of its components
❖ WOCBP must have a negative urinary pregnancy test the day of inclusion	❖ WOCBP without contraceptive method, or with positive pregnancy test, breastfeeding, or planning to become pregnant during the study period
❖ All sexually active male subjects must agree to use an adequate method of contraception throughout the study period and for 90 days after the last dose of study drug and agree to no sperm donation until the end of the study, or for 90 days after the last dose of XAV-19, whichever is longer	❖ Current documented and uncontrolled bacterial infection
❖ Patients with French social security	❖ Prior severe (grade 3) allergic reactions to plasma transfusion
	❖ Patient participating in another interventional clinical trial
	❖ Life expectancy estimated to be less than 6 months
	❖ Patient under guardianship or trusteeship

### Who will take informed consent {26a}

Patient’s written consent will be obtained by the investigator prior to any study-specific procedures. Participation is voluntary, individuals may withdraw at any stage, and participation does not affect their treatment.

### Additional consent provisions for collections and use of participant data and biological specimens {26b}

We will take advantage of the existing “Immunology” research program declared by CHU Nantes to set up the “POLYCOR” biocollection. Biological samples will be collected and stored if the patient has signed the biocollection informed consent. In this informed consent form, it is noted that the samples may be used for other scientific research. This biocollection will be a biological and longitudinal follow-up of patients with diagnosis of COVID-19, to assess immune changes and host-virus interactions. Ethic authorization is already granted, and the preanalytical steps are already implemented and efficient.

### Interventions

#### Explanation for the choice of comparators {6b}

POLYCOR project has the aim to define the optimal XAV-19 dose to administrate to moderate COVID-19 pneumonia patients, and to evaluate its safety and efficacy.

French regulatory authorities (ANSM), thus, has given their scientific recommendation to conduct the first part of the study (phase 2a) as a first-in-human study, with sequential enrolment to check for safety.

Dose levels selected in the first-in-human assessment of XAV-19 are based on 3 datasets:

1. Clinical experience with LIS1 glyco-humanized swine IgG administration. LIS1 is an experimental lymphodepleting polyclonal swine antibody produced by the glyco-humanized GH-pAb platform of Xenothera Laboratory. It is currently in clinical development in kidney transplant patients in EU (EUDRACT 2019-000917-35). So far, the maximal dose that has been reached in the escalating dose arm and found acceptable by the DSMC (which recommended pursuing the trial at this dose) is five daily administrations of 8 mg/kg, i.e., a cumulated dose of 40 mg/kg. Therefore, in this first-in-human trial with XAV-19, we have proposed that the cumulative dose stays inferior to the maximal LIS1 dose administered.
2. Published clinical experience gained with use of convalescent plasma (CP) containing anti-COVID-19 neutralizing antibodies, having evidenced improvement of clinical symptoms. First, critically ill patients with COVID-19 received two consecutive transfusions with 200 mL CP. CP had a SARS-CoV-2-specific antibody (IgG) binding titer greater than 1:1000 and a neutralization titer greater than 40 (end point dilution titers) [9]. In this experience, the ratio between binding and neutralization titers is therefore of 25-fold on the average. Second, Duan et al. treated 10 COVID-19 patients with one dose of 200 mL CP [21]. In this report, the average neutralizing titer calculated on 40 CP was > 1/160. Third, in a former clinical experience with 80 CP transfusions from SARS-convalescent patients in 2003, the average CP volume was 279 mL and the neutralizing titer was > 1/160 [22]. Thus, all together, estimating that transfusion of 200 mL CP corresponds to 25 mg/kg IgG (considering that plasma contains 10 mg/mL IgG and considering 80-kg patients), we can postulate that 25 mg/kg of an IgG preparation presenting a neutralizing titer of at least 1/40 presents a clinical interest. Binding and neutralization titers of glyco-humanized serum from which XAV-19 has been obtained were of approximately 1/100,000 and 1/4000 (in the ELISA neutralization assay). The neutralization titer in a cytopathogenic effect assay was of 1:1600. This neutralizing titer of XAV-19 is thus 40- to 100-fold higher (depending on the assay) as compared with COVID-19 CP presenting a clinical interest according to the literature, and 12.5-fold higher than recommended by the European Commission. It has been considered that the ratio of the neutralization titer in XAV-19 vs CP should be used in the dose selection. According to available data, a batch exhibiting a 40-fold ratio to CP might thus be used at a 40-fold inferior dose, which means that infusions of 0.6 mg/kg XAV-19 should result in similar effects in vivo as compared with 25 mg/kg CP. Recent guidance from the European Commission on use of CP revised desired neutralizing titers of CP and suggested that “neutralizing antibody titers should optimally be greater than 1:320, but lower thresholds might also be effective” (https://www.phc.org.ua/sites/default/files/users/user90/Guidance_plasma_covid19_en.pdf). Therefore, another, higher dose than 0.6 mg/kg XAV-19 might also be tested.
3. PK/PD information obtained from ex vivo assessment of XAV-19. XAV-19 has been assessed in competition ELISA (inhibition of the COVID-19 spike protein binding to ACE-2 receptor) and tested in neutralizing activity assay against SARS-CoV-2 using classical plaque reduction test using Vero cells. Our data showed an IC_50_ (concentration for reaching 50% of maximal signal in the test) of approx. 2.5 μg/mL of the first batch of XAV-19 in these ELISA and neutralization assays [14]. Data from the cytopathogenic effect assay showed a complete inhibition of SARS-CoV-2 infectivity was reached around 15–25 μg/mL in the assay. Considering an average plasma volume of 38.5 mL/kg, infusions of 0.96 mg/kg of this batch of XAV-19 would be required to reach (at Cmax) 25 μg/mL plasma concentration (at Cmax). The volume of distribution of swine glyco-humanized IgG measured in primates (53 mL/kg) has a value close to that of the plasma suggesting that the XAV-19 IgGs will essentially circulate in the plasma. Considering also distribution and elimination half-life, estimated at 172 h in primates, we can select an initial dose 2-fold higher than this target, given on day 1 and day 5, so as to stay above a through level of 25 μg/mL over more than a week, a duration deemed appropriate to obtain a clinical response. The estimated dose to reach this target is 2 mg/Kg.

Based on these 3 datasets, the Pharmacologically Active Dose (PAD) Standard of Care SOC for Group 2 of phase 2a would be 2 mg/kg infused on day 1 and on day 5 for a batch of XAV-19 presenting the IC_50_ of 2.5 μg/mL in the ELISA Spike/ACE2 neutralization assay. It is meant to maintain trough levels of XAV-19 above 25 μg/mL over several days. This dosing is lower than the dose already administered in human in the previous clinical experience demonstrating safety with LIS1 (5 daily administrations of 8 mg/kg, thus a total of 40 mg/kg), thus lower than a dosing which might reach a dose limiting toxicity. In other words, this dosing (two administrations of 2 mg/kg) presents a 10-fold safety factor for the first administration of XAV-19, as compared with previous experience with glyco-humanized swine IgG. Based on this PAD, the first-in-human dose (group 1 of phase2a) can be set with a safety factor of four following the escalation guidelines of the European Commission. For the first-in-human dose, 0.5 mg/kg on day 1 and on day 5 is therefore proposed, i.e., a cumulated dose of 1 mg/kg. Thus, there is a 4-fold increment between group 1 and group 2 in the phase 2a.

The evaluation of safety and phamacokinetic parameters in both dosing groups will guide the choice for the dose to be used in the phase 2b study.

#### Interventions description {11a}

Phase 2a will enroll 16 subjects in one of the two dose-level groups: group 1 with dose level defined according to the neutralization titer of the XAV-19 batch (first estimation being 0.5 mg/kg) and group 2 as 4 times higher than group 1 (estimated 2.0 mg/kg). If a patient does not receive the full treatment or has not been followed for 8 days, then he/she will be replaced within the limit of 20 patients in total. Each subject will receive two infusions of XAV-19 or placebo separated by an interval of 4 days (administrations on day 1 and day 5).

More precisely, after enrollment of the first two patients in the low dose group (one patient treated and one patient placebo), data will be obtained after first (day 1) and second (day 5) infusions to collect any severe adverse events that might have occurred, especially in the first 48 h following each infusion.

DSMC will have to review immediately safety data of the first 2 patients (based on day 8 information) and provide advice on continuing enrolment of the 6 last patients of group 1 (low dose). DSMC statement, and sponsor decision with regard to study continuation will be immediately communicated to ANSM.

If there is no signal of severe intolerance by day 8 of enrollment of the first two patients, the rest of the group will be enrolled with the same process of review of tolerance.

When the final patient of group 1 has been enrolled, day 8 safety data of all 8 patients from this low-dose cohort will have to be reviewed by the DSMC to advise of starting high-dose group (group 2). DSMC statement and sponsor decision with regard to study continuation will be immediately communicated to ANSM.

At any time during the phase 2a study, if a serious adverse event occurs, this will be communicated to the DSMC and the sponsor and coordinating investigator will have the responsibility to decide whether DSMC must meet to advise on study conduct.

The same sequential XAV-19 administrations will apply for the second group dosed at 2 mg/kg, with safety steps and DSMC data review.

At the end of phase 2a and as requested by the French regulatory authorities (ANSM), an amendment will be made to the protocol to continue the study in phase 2b.

The phase 2b (DFD) will be a double-blinded, placebo-controlled randomized study to assess clinical benefit and safety of the phase 2a selected dose of XAV-19 in 352 hospitalized adults with COVID-19-associated moderate pneumonia. Patients will be randomized to either XAV-19 or placebo in a 1:1 ratio and will receive 2 consecutive doses 4 days apart (day 1 and day 5). Obviously, during this 2b study, the DSMC will regularly meet as defined in the DSMC charter.

During the phase 2b study, if the proportion of patients requiring invasive mechanical ventilation after inclusion increases to 30~50% (analyzed by incremental groups of 50 patients), the DSMC will be requested to review data without discontinuation of the study. If the proportion of patients requiring invasive mechanical ventilation after inclusion is greater than 50% of the patients included in the study (analyzed by incremental groups of 50 patients), inclusions will be temporarily stopped and DSMC will be requested to review data and decide on an early definitive discontinuation of the study.

**Fig. 1:**
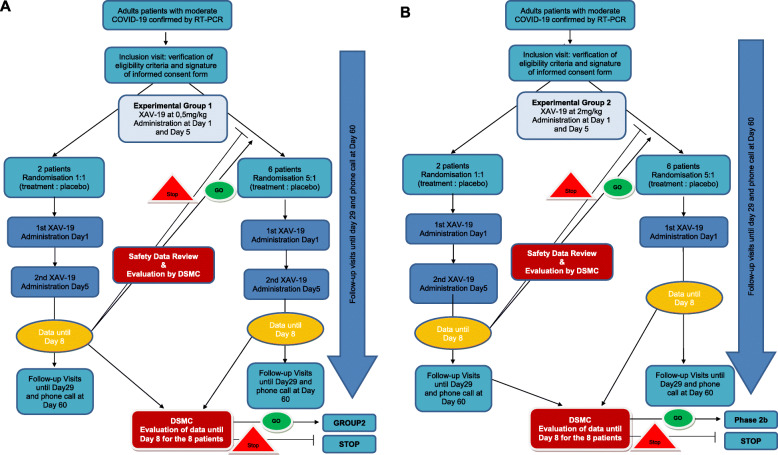
Diagram of phase 2a study. **a** Phase2a—group1. **b** Phase 2b—group 2

**Fig. 2:**
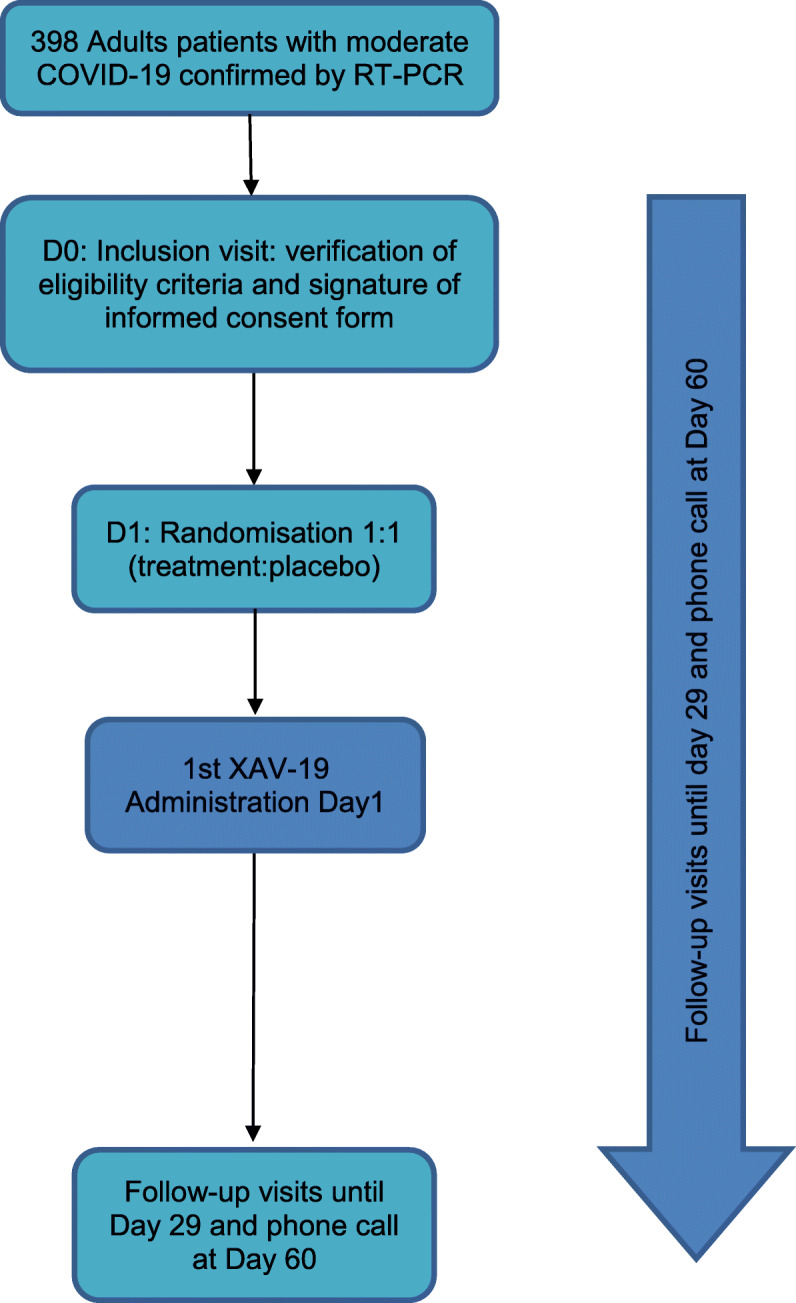
Diagram of phase 2b study

The study diagram of these 2 studies are showed in Figs. 1 and 2.

**Fig. 3:**
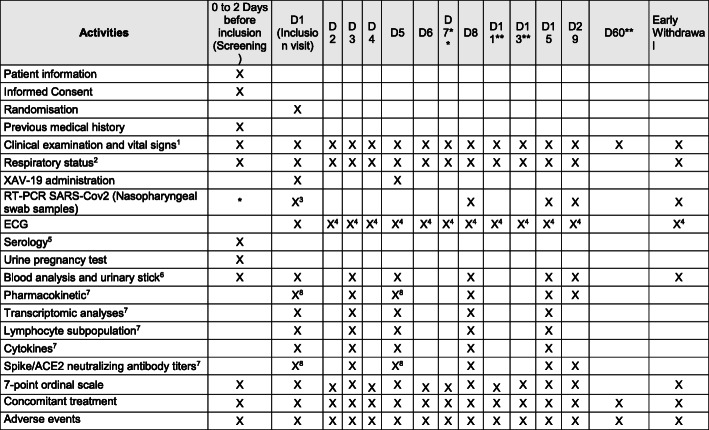
Study schedule for phase2a and phase 2b

The study schedule with all the visits is summarized in the flowchart showed in Fig. 3; all of these examinations apply to all patients from phase 2a and phase 2b, except for the specific blood samples specific to ancillary studies. In phase 2a, the blood collections apply for all patients. In the phase 2b, the following blood collections are part of ancillary studies as follows:

Ancillary study 1: 20 patients to evaluate the pharmacokinetic

- ▪ Lymphocyte sub-populations

Ancillary study 2: 30 patients from phase 2b to evaluate:

- ▪ Spike/ACE2 interaction neutralizing antibody sample
- ▪ Lymphocyte sub-populations
- ▪ Cytokines
- ▪ Transcriptomic analyses
- ▪ Single cell RNAseq on B cells
- ▪ B-Tfh functional interaction

#### Criteria for discontinuing or modifying allocated interventions {11b}

In the event of early discontinuation of the study treatment, all efforts will be made by the investigator to invite the patient to undergo the end of visit. The primary reason for discontinuation must be recorded in the appropriate section of the e-CRF and all efforts made to complete and report the observations as thoroughly as possible.

If a patient fails to return for a scheduled visit/follow-up, attempts should be made to contact the patient to identify reason for not returning. Likewise, if a patient declares their wish to discontinue from the study, e.g., for personal reasons, all efforts should be made to establish that the cause is not due to an AE (bearing in mind the patient is not obliged to their reasons).

A definitive or temporary discontinuation of all or part of the study may be decided by ANSM, the Ethics Committee, the Sponsor after Data and Safety Monitoring Committee (DSMC) opinion*.*

In any case:

- A written confirmation of this early discontinuation of the study shall be sent to the coordinating investigator of the study (specifying the reasons for the early discontinuation).
- All the patients included in the study shall be informed and should attend their early withdrawal visit.

Each early departure from the study should receive the usual medical follow-up.

#### Strategies to improve adherence to interventions {11c}

The studies will be realized in hospital; patient adherence is not applicable.

#### Relevant concomitant care permitted or prohibited during the trial {11d}

SOC for COVID-19 will be conducted per local practice and/or local/national guidelines at time of the study, which may include, not exclusively, antibiotics, antiviral treatment, corticosteroids, immune therapies not based on antibodies administration, and anticoagulants. Specifically, serotherapy for the current COVID-19 episode will not be permitted.

#### Provisions for post-trial care {30}

After the end of the study, each patient will receive the usual medical follow-up.

The sponsor takes out an insurance policy covering the financial consequences of its civil liability in compliance with the regulations.

### Outcomes {12}

The primary endpoints of the phase 2a study are the (a) pharmacokinetic measurement of the antibody titer of XAV-19 measurements of all treated patients and of all patients in the placebo group at day 8 (3 days after the last administration) and (b) tolerability and adverse events of XAV-19 between the two groups of treated patients and vs. placebo over 29 days, evaluated as:

- Occurrence of all suspected XAV-19-related adverse effects and incidence of serious adverse events
- Proportion of participants with treatment emergent adverse events leading to study drug discontinuation
- Incidence of major or bacterial or fungal infections during the hospital stay
- Incidence of hypersensitivity reactions and infusion reactions. To note AEs are registered up to day 60 and for infusion-related AEs, 48 h after.
- White cell count, hemoglobin, platelets, creatinine, ALT, AST, on day 1, day 3, day 5, day 8, day 15, and day 29
- SARS-CoV-2 viral load over time (day 1–day 29), as collected by nasopharyngeal swab samples
- Time to RT-PCR virus negativity in nasopharyngeal swab samples

The secondary endpoints of the phase 2a are the:

- Pharmacokinetic analysis: Antibody titer measurements at day 1 (pre-dose, post-dose), at day 3, day 5 (pre-dose, post-dose), day 8, day 15, and day 29.
- The antibody titer of XAV-19 measurements in group 1 treated patients and group 2 treated patients at day 15.
- Clinical aspects:

- Duration of supplemental oxygen
- Transfer to intensive care unit with need for invasive mechanical ventilation or high flow oxygen
- Normalization of fever ≥24 h: clinical assessment every day from day 1 to day 14. Evaluation to be performed between 8 and 12 am, day X evaluation will consider the higher value during day X-1 (8 am to 8 am)
  - Normal fever (≤ 37.8 °C tympanic or ≤ 36.6 °C axillary or ≤ 37.2 °C oral taken 4 h apart from antipyretic administration) each day and time to resolution of fever for at least 48 h
- Biomarkers: CRP, Ferritin (at days 1, 3, 5, 8, 15, and 29)
- Ordinal scale assessed at Day 15. The 7-point ordinal scale is an assessment of the clinical status at the first assessment on day 15. The scale is as follows:

1. Not hospitalized, no limitations on activities
2. Not hospitalized, limitations on activities
3. Hospitalized, not requiring supplemental oxygen
4. Hospitalized, requiring supplemental oxygen
5. Hospitalized, on non-invasive ventilation or high flow O_2_ devices
6. Hospitalized, on invasive mechanical ventilation or ECMO
7. Death

- Hospital length of stay

The primary endpoint of the phase 2b is the time to weaning of supplemental oxygen.

If patient is still on oxygen at day 15 or if the patient is weaned but put back on oxygen, then the delay will be censored at 15 days.

The secondary endpoints are the:

- National Early Warning Score (NEWS2) at day 15 and difference in NEWS2 between day 1 and day 15
- Clinical status using the 7-point ordinal scale assessed at day 3, day 5, day 8, day 11, day 15, and day 29
- Time to improvement of one category from admission using the 7-point ordinal scale
- Time to first fever normalization (criteria for normalization: temperature < 36.6 °C armpit, < 37.2 °C oral, < 37.8 °C rectal or tympanic)
- Duration of oxygen therapy over 29 days
- Comparison of oxygen requirement between the two groups over 29 days
- Time to weaning in supplemental oxygen and proportion without O2 requirement at day 15, according to baseline (day 1) oxygen requirement (≤ 4 L/min or 4 L/min)
- Incidence and duration of non-invasive ventilation or high flow oxygen devices, of invasive mechanical ventilation during the study
- Hospital length of stay
- All-cause mortality at Day 29
- Safety of XAV-19 evaluated as:

- Occurrence of all suspected XAV-19-related adverse effects or incidence of serious adverse events
- Proportion of participants with treatment emergent adverse events leading to study drug discontinuation
- Incidence of major or opportunistic bacterial or fungal infections
- Incidence of hypersensitivity reactions and infusion reactions
- White cell count, hemoglobin, platelets, creatinine, ALT, AST, on day 1, day 3, day 5, day 8, day 11, day 15, and day 29
- SARS-CoV-2 viral load over time (day 1–day 29), as collected by nasopharyngeal swab samples
- Time to RT-PCR virus negativity in nasopharyngeal swab samples

### Participant timeline {13}

For the phase2a study: The treatment duration per patient will correspond to the infusion made at day 1 and day 5, the patient’s follow-up to 2 months, and the recruitment period to 2 months.

For the phase2b study: The treatment duration per patient also will correspond to the infusion made at day 1 and day 5, the patient’s follow-up to 2 months, and the recruitment period to 6 months.

### Sample size {14}

As it is a phase 2a, the number of patients was determined according to phase 2a’s relevant publications [23–25] . It was decided to enroll two cohorts of 8 patients (6 treated patients and 2 matching placebo in each cohort).

For the phase 2b, the predetermined sample size, controlling for the α risk of 5% and power of 80%, is 352 participants, based on an expected survival rate of patients giving up oxygen therapy at least at day 15 of 80% in the control group and 90% in the experimental group. In addition, the median duration of oxygen therapy in the control group is 8 days, and in the experimental group is 6 days. Hence the estimated hazard ratio is 0.75. The sample size was calculated following the formula of Schoenfeld [26] and the hazard ratio was estimated thanks to Cortes’ publication [27].

### Recruitment {15}

Recruitment is planned over a period of 4 months for the phase 2a study and 6 months for phase 2b study. The number of open investigator centers compensates for the short duration of inclusion.

## Assignment of interventions: allocation

### Sequence generation {16a}

Randomization will be performed and balanced by blocks. The software used for the randomization is SAS version 9.4.

In terms of the ratio, for phase 2a, the first 2 patients in each cohort will have a ratio of 1:1, and if all goes well, the remaining 6 patients in the 2 cohorts will have a ratio of 1:5 (1 placebo for 5 doses of XAV-19). For the phase 2b, it will be carried out in a 1:1 ratio.

### Concealment mechanisms {16b}

The randomization will be carried out via EnnovClinical software by connecting to the website: https://nantes-lrsy.hugo-online.fr/. The connection will be made using a login, a password, and a study number, issued by a data manager of the Sponsor Department of CHU Nantes. The inclusion number and arm of randomization will be assigned automatically.

### Implementation {16c}

The randomization key is known only to the biostatistician, who will not participate to the study, and the data managers, to make it impossible for the investigator to assign a particular treatment.

### Assignment of interventions: blinding

#### Who will be blinded {17a}

Our studies are randomized double-blinded trials. The patients and the investigators and their teams will be blinded and will not know the treatment assigned. As Karanicolas et al. pointed out in their article from 2010, their study should have been masked to the biostatisticians until analyses had been performed, to reduce the study bias [28].

#### Procedure for unblinding if needed {17b}

Under normal circumstances, blind should not be broken until all patients have completed the study and the database is locked. In the current study, the investigator does not need to know the treatment arm of the patient in case of adverse event, as the treatment will be symptomatic and not adapted to the arm. The blind will be broken only by the pharmacovigilant team for safety purposes and transmission. This will be done according to the internal sponsor’s procedure.

## Data collection and management

### Plans for assessment and collections of outcomes—description of the parameters for evaluating efficacy {18a}

All examinations will be performed by the investigator or a qualified member of the investigational staff. Firstly, he will make a full physical examination which includes the following assessments: cardiovascular, head-ear-nose-throat, eyes, gastro-intestinal, general appearance, lymph nodes, musculoskeletal, neurological, respiratory, skin, and mucous membranes. Body weight and height will be measured.

Any abnormalities present at inclusion, or subsequent changes, will be documented in the appropriate sections of the eCRF. Any clinically significant abnormalities persisting at the end of the study will be followed by the investigator until resolution or until reaching a clinically stable endpoint. Vital signs and respiratory status will be reported. A 12-lead digitalized ECGs will be recorded according to the study schedule while the patient is in supine position for at least 5 min. ECG is performed at inclusion visit; other ECG will be performed at the discretion of the investigator, as deemed necessary for appropriate patient care.

Clinical patient status will be assessed by a seven-level ordinal scale (with levels ranging from one to seven and higher scores indicating a worse condition). This scale will be assessed at each study visit. Each day, the worse score for the previous 24 h will be recorded, i.e., on day 3, day 2 score is obtained and recorded as day 2. On day 1 and day 5, assessment will have to be performed before XAV-19 infusion.

We will use also NEWS2 which is the latest version of the National Early Warning Score (NEWS), updated in December 2017 [20], which advocates a system to standardize the assessment and response to acute illness. NEWS2 is based on a simple aggregate scoring system in which a score is allocated to physiological measurements, already recorded in routine practice, when patients present to, or are being monitored in hospital. Six simple physiological parameters form the basis of the scoring system: (1) respiration rate, (2) oxygen saturation, (3) systolic blood pressure, (4) pulse rate, (5) level of consciousness or new confusion, and (6) temperature. A score is allocated to each parameter as they are measured, with the magnitude of the score reflecting how extremely the parameter varies from the norm. The score is then aggregated and uplifted by 2 points for people requiring supplemental oxygen to maintain their recommended oxygen saturation.

### Plan to promote participant retention and complete follow-up {18b}

As a reminder, the target population will be hospitalized; missing data could only result of withdrawal of the consent or a serious adverse event (SAE). No plan to promote participant retention has been realized.

### Data management {19}

For each patient, a case report form (CRF) is created which includes the data necessary to ensure compliance with the protocol and all data necessary for the statistical analysis and to identify major protocol deviations. Data collection is done directly by the investigator or clinical research associate in charge of the study, using an electronic CRF (eCRF) developed by the Sponsor Department of the CHU Nantes with ENNOV Clinical. The data are encoded to keep the identities of the patients confidential.

### Confidentiality {27}

Data collected during the study will be processed electronically in compliance with the requirements of the CNIL (compliance with the French Reference Methodology MR001). The CNIL is an independent French administrative regulatory body whose mission is to ensure that data privacy law is applied to the collection, storage, and use of personal data.

### Plans for collection, laboratory evaluation, and storage of biological specimens for genetic or molecular analysis in this trial/future use {33}

For the pharmacokinetics analysis, blood samples will be collected at each draw for specific analysis as described below according to the study schedule. These analyses will be run under Good Laboratory Practices conditions. In the phase 2a, all patients included in the study will have pharmacokinetic samples taken.

For the immunological analyses, blood samples will be collected for specific analysis as described below according to the study schedule. Actual time and date of blood drawn must be accurately recorded on the eCRF.

The following analyses will be performed:

- T and B lymphocyte sub-populations (spectral flow cytometry)
- Tfh-B cell interaction
- Transcriptomic analysis on PBMC and B cells
- Spike/ACE2 interaction neutralizing antibody
- Serum cytokines: MCP1, IP10, IL-10, IFNα, TNF-α, IL-1b, IL-6, IL-82 (in the phase 2a) and IL-6, IFNa2, IL-10, IP10, CCL2 (in the phase 2b)

Finally, the transcriptomic analyses of messenger RNA molecules will be extracted from peripheral blood in order to identify transcripts that are specifically over- or under-expressed in correlation with the clinical status and with viral load. The goal is to identify possible biomarkers of the disease severity and disease responding to the treatment and, using deconvolution bioanalyses, also identify cell population changes in complement to the direct flow cytometry analyses. A focus will be made on B cell sub-populations and their role in interaction with Tfh cells. These immunological analyses will be performed by the CRTI in Nantes University (INSERM U1064) (led by Sophie Brouard, CNRS).

At the end of the study, residual biological samples resulting from sampling (blood sample) for all patients from phase 2a and only for patients accepting to participate to the immunomonitoring study in the phase 2b will be kept. In the latter case, the subject’s written consent will be collected at screening and the samples stored will be integrated into the biocollection “POLYCOR” located in the Centre de Ressources Biologiques (CRB) of the CHU Nantes, under the responsibility of Dr. Gaborit. This biocollection will be attached to the “Immunologie” research program declared on 05/09/2011 under the no. DC-2011-1399 and in the following amending declarations (DC-2012-1555; DC-2013-1832, DC2014-2206, and DC-2017-2987 currently pending) at the Ministry of Research and having obtained a favorable decision from the CPP Ouest VI (Ethics Committee) on 7 April 2015.

## Statistical methods

### Statistical methods for primary and secondary outcomes {20a}

For the phase 2a study, the safety population included all subjects randomized into the study who received at least one dose of study drug. The intention-to-treat (exposed, ITT[E]) Population was defined as all subjects who met study criteria and were randomized into the study with documented evidence of having received at least one dose of randomized treatment and at least one post-baseline XAV-19 IgG SARS-CoV2 titer measurements. The Per-Protocol Population was defined as all subjects included in the ITTE Population excluding those who had at least one major protocol deviation.

The primary endpoint will be evaluated for ITTE Population by a Kruskal-Wallis test between all placebo patients and all treated patients.

Groups of treatment will be described according to variables in of the secondary endpoints. Categorical variables will be summarized by percent and continuous variable by means and standard error, and medians and interquartile range.

For the phase 2b, categorical variables will be summarized by percent and continuous variable by means and standard error, and medians and interquartile range. Survival rate and duration of oxygen therapy will be compared between the two groups for the primary endpoints.

For the secondary endpoint, quantitative endpoint will be compared using linear mixed model and qualitative data with logistic mixed model to take into account stratification factors. Time data’s will be analyzed using survival model. Fine and Gray method will be used to take into account competing risk.

### Interim analyses {21b}

No interim analysis will be performed and no early stopping rule for futility will be proposed.

### Methods for additional analyses (e.g., subgroup analyses) {20b}

The PK Concentration Population included all subjects who received XAV-19 at least once during the study. The PK Summary Population included subjects with an evaluable pharmacokinetic profile of XAV-19 on day 8. The PK/PD Summary Population included subjects who met the criteria for both PP Population and PK Summary Population.

The pharmacokinetic analysis will be evaluated by the study of the accumulation, time-invariance, achievement of steady state, and dose-proportionality by ANOVA models with terms for subjects as a random effect and day as fixed effect.

### Methods in analysis to handle protocol non-adherence and any statistical methods to handle missing data {20c}

For phase2a study, no imputation will be realized in this study. Patient who did not receive both doses or followed up until D8 will be replaced. For phase 2b, we do not expect missing data for the primary outcome. However, if patient is followed up then data will be censored at last known date. No imputation will be used for secondary efficacy and safety outcomes. No subject replacement is planned.

### Plans to give access to the full protocol, participant-level data, and statistical code {31c}

According to French law, the results of the study will be published on the website of the regulatory authority. Data sharing will be between the investigators only. However, the datasets analyzed during the current study will be available from the corresponding author on reasonable request. Furthermore, the sponsor will enter the study results in the European Union database as soon as the main publication from the research is released, in order to preserve intellectual property.

## Oversight and monitoring

### Composition of the coordinating center and trial steering committee {5d}

It has been possible to carry out the protocol and the trial thanks to a Scientific Committee created and coordinated by Dr. B Gaborit. The Scientific Committee’s missions are:

- Approval of DSMC composition
- To ask for information regarding the progress of the research project, any potential issue, and available results
- To ensure compliance with ethics requirements
- To perform the scientific follow-up of the research: maintain the relevance of the research objectives and the permanent validity of the methods implemented to meet them
- To make all important decisions at the demand of the Coordinating Investigator or the DSMC regarding the good conduct of the research in compliance with the protocol, any procedure specific to the research, and Good Clinical Practices
- To decide on all relevant modification of the protocol required to achieve the research project (including recruitment facilitating measures, protocol amendments before regulatory submissions, addition or closure of participating sites)
- To provide information to all investigators and other participants in the research,
- To ensure that rules related to data and biological samples access are followed,
- To ensure that rules related to the communication and publication of research results are fulfilled

Meeting minutes are drafted following each meeting by the Project Leader, together with the president of the Scientific Committee (at least an exhaustive list of the issues discussed and the decisions made as well as the list of the points raised). The minutes are submitted for review and modifications to the members of the Scientific Committee present at the meeting. The minutes are then validated by the president of the Scientific Committee. It is then distributed to the Scientific Committee members and to the persons invited to the meeting, as well as to the Head of the Nantes Hospital clinical research department. The minutes are definitely adopted at the beginning of the following meeting of the Scientific Committee.

### Composition of the data monitoring committee, its role and reporting structure {21a}

The role of the DSMC is to review the progress of the trial and the accumulating data to detect evidence of early safety issues for the enrolled subjects. The DSMC will give recommendation regarding modification to the ongoing conduct of the trial (see Figs. 1 and 2) and give approval for continuing the study to the next step within each group of phase 2a and between the phase 2a and 2b.

The competent members in the field of clinical trials (disease and methodology) are not involved in the study. They are appointed for the period of the study and undertake to participate and to respect the data confidentiality.

The annual safety report is sent to the Data and Safety Monitoring Committee. The committee may be requested for a review by the person in charge of safety pharmacology if a SUSAR or a SAE presents a particular analytical problem or if a doubt in respect benefit/risk arises during the study.

### Adverse event reporting and harms {22}

All patients will be closely monitored during both study phases for overall biological and clinical safety data (e.g., infections or malignancies) and any drugs toxicity.

According to regulation, each AE reported by the patient or identified by the investigator must be collected and reported to sponsor, as soon as he is aware, if it meets to seriousness criteria from inclusion of the subject, to the end of the participation.

As a routine precaution, patients enrolled in this trial must be observed for at least 2 h post infusion of study drugs. Patients will be assessed for signs or symptoms of any infusion reactions (e.g., hypotension, hypoxia, tachycardia, fever, nausea, fatigue, headache, myalgia, and malaise).

### Frequency and plans for auditing trial conduct {23}

An inspection or audit may take place as part of this study, performed by the sponsor or/and by the regulatory authorities. Inspectors will check the documents, logistics, records, and any other resources that the authorities consider to be associated with the clinical trial and that may be located at the trial site itself.

### Plans for communicating important protocol amendments to relevant parties (e.g., trial participants, ethical committees) {25}

The amended protocol will be a dated, updated version. If necessary, the information form and consent form should be amended. The sponsor project manager will notify the centers and a copy of the revised protocol will be send to all the principal investigators to add to the Investigator Site File. Currently, the updated protocol is at version 1.2 on 17 July 2020. All the submissions/declarations will be made by the Sponsor Department at CHU Nantes to the French regulatory authority (ANSM) and the ethic committee (Comité de Protection des Personnes Ouest VI). The clinical research associate (CRA) of the Sponsor Department will report any deviations of the protocol. They will be fully documented using a breach report form.

### Disseminations plans {31a}

The results of this study will be published or presented at scientific meetings.

## Discussion

This study will be the first randomized controlled trial to evaluate pharmacokinetic, pharmacodynamic, safety, and efficacy of polyclonal humanized anti-SARS-CoV2 antibodies during moderate pneumonia induced by COVID-19.

Although most patients infected with SARS-CoV-2 have a mild illness, over 25% of hospitalized patients for COVID19 disease develop acute respiratory distress during the second week of hospitalization with the onset of severe ARDS. Passive antibody therapy is a promising treatment strategy, involving administration antibodies to confer immediate immunity to neutralize virus invasion.

For SARS-CoV-1, high mortality without any specific therapy has led to passive immunotherapy using convalescent plasma [29]. The early delivery of convalescent plasma in patients without immunity seemed associated with better results, particularly for patients receiving therapy during the first 14 days of disease [22].

In COVID-19, first reports of convalescent plasma to treat hospitalized patients suggested a potential efficacy in the most severe forms with acceptable tolerability [19] and safety evaluated among more 5000 treated patients [30]. Recent reports suggesting convalescent plasma may be beneficial in the initial stage of COVID-19 led by IDSA to support further data collection in randomized clinical trials to better assess its benefits.

However, difficulties to obtain plasmas containing enough neutralizing antibodies lead to the development of innovative treatments based around a similar approach as for the “XAV-19”. The active ingredient of XAV-19 is a stabilized solution of purified hyper immune anti-SARS-CoV-2 spike RBD protein swine glycol-humanized IgG with neutralizing activity. Indeed, polyclonal humanized anti-SARS-CoV2 has the potential to significantly decrease the severe complications induced by SARS-CoV-2 in patients by effectively freezing the disease process. Other benefits are to blunt the pneumopathy-induced damage and other COVID-19-associated injuries such as acute kidney injury (AKI), myocarditis, and secondary bacterial infections. This treatment is expected to shorten the duration of requirement for oxygenotherapy and of hospital stay with minimization of physical, psychological, and economic complications related with prolonged stay.

The XAV-19 IgGs are being produced from genetically modified pigs and present the following characteristics and advantages, as compared to existing polyclonal antibodies: (1) glyco-humanization, allowing a low native immunogenicity [15]; (2) augmented complement-mediated cytotoxicity (CDC) [31]. Indeed, glyco-humanized swine IgG antibodies have a 3D structure of their Fc fragments that allows increased capacities to recruit human C1q proteins and to activate complement; (3) absence of possible antibody-dependent enhancement (ADE), due to the absence of binding to human Fc receptors [32]. ADE refers to enhanced infectivity of a microorganism by allowing Fc receptor-dependent entry into Fc receptor positive cells (mainly macrophages); (4) binding to SARS-CoV-2 spike molecule; and (5) neutralizing activity against SARS-CoV-2.

According to the preclinical data, the anticipated clinical benefit of XAV-19 in comparison with existing antibody therapies (so far limited to transfusions on convalescent plasma) may be an increased efficacy and a better tolerance relative to potential other treatments with heterologous polyclonal antibodies (in development).

## Trial status

The recruitment period for phase 2a has started on 1 September 2020 with the inclusion of the first patient. Phase 2a was completed at the end of December 2020 and phase 2b has started on January 2021.

The updated protocol is at version 1.4 on 4 August 2020.

## Supplementary Information

**Additional file 1.**

## Abbreviations

ACE2: Angiotensin-converting enzyme 2
ADE: Antibody-dependent enhancement
AE: Adverse event
ANSM: Agence Nationale de Sécurité du Médicament et des produits de santé – French regulatory authorities
ARDS: Acute respiratory distress syndrome
CNIL: Commission Nationale de l’Informatique et des Libertés – French National Commission on Informatics and Liberty
CP: Convalescent plasma
CRA: Clinical Research Associate
CRF: Case report form
DAD: Double ascending dose
DFD: Double fixed dose
DSMC: Data and Safety Monitoring Committee
eCRF: Electronic case report form
ITTE: Intention to treat exposed
NEWS: National Early Warning Score
PAD: Pharmacologically Active Dose
PD: Pharmacodynamic
PK: Pharmacokinetic
SAE: Serious adverse event
SAR: Serious adverse reaction
SARS: Severe acute respiratory syndrome
SOC: Standard of care
SUSAR: Suspected unexpected serious adverse reaction
WOCBP: Women of childbearing potential
