# Supplementary material for: Evaluation of the safety and efficacy of XAV-19 in patients with COVID-19-induced moderate pneumonia: study protocol for a randomized, double-blinded, placebo-controlled phase 2 (2a and 2b) trial
Source: Trials. 2021 Mar 9;22:199. doi: 10.1186/s13063-021-05132-9 (PMC7942514; doi:10.1186/s13063-021-05132-9)
Supplement: Supplementary file 1 — Additional file 1. [file 13063_2021_5132_MOESM1_ESM.doc]

***
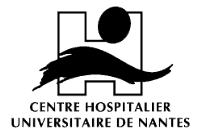
***

**Note d’information pour la participation à la recherche**

**Phase 2a**

**«**Etude de phase 2 (2a et 2b) randomisée en double aveugle, contrôlée par placebo pour évaluer la sécurité et l'efficacité XAV-19 chez des patients atteints d’une pneumonie modérée à COVID-19 **»**

**Titre abrégé : « POLYCOR»**

***Promoteur : CHU de Nantes***

***N° EudracT/ Enregistrement :*** 2020-002574-27

# SOMMAIRE

[SOMMAIRE 1](#__RefHeading___Toc44314026)

[INFORMATION SUR LE PRODUIT A L’ETUDE 2](#__RefHeading___Toc44314027)

[BUT DE LA RECHERCHE 2](#__RefHeading___Toc44314028)

[DEROULEMENT DE LA RECHERCHE 3](#__RefHeading___Toc44314029)

[RISQUES DE LA RECHERCHE 4](#__RefHeading___Toc44314030)

[BENEFICES ATTENDUS DE LA RECHERCHE 5](#__RefHeading___Toc44314031)

[ALTERNATIVES A LA RECHERCHE 5](#__RefHeading___Toc44314032)

[FIN DE LA RECHERCHE 5](#__RefHeading___Toc44314033)

[FRAIS SUPPLEMENTAIRES 6](#__RefHeading___Toc44314034)

[VOS DROITS PENDANT LA RECHERCHE 6](#__RefHeading___Toc44314035)

[VOS OBLIGATIONS PENDANT LA RECHERCHE 7](#__RefHeading___Toc44314036)

[LE CADRE REGLEMENTAIRE 7](#__RefHeading___Toc44314037)

[DEVENIR DE VOS ECHANTILLONS PRELEVES AU COURS DE LA RECHERCHE 7](#__RefHeading___Toc44314038)

[VOS CONTACTS : 8](#__RefHeading___Toc44314039)

[EN RESUME… 9](#__RefHeading___Toc44314040)

[ANNEXE 1 : CALENDRIER DES VISITES DE LA RECHERCHE 10](#__RefHeading___Toc44314041)

[ANNEXE 2 : CRITERES D’INCLUSION ET D’EXCLUSION DE L’ETUDE 11](#__RefHeading___Toc44314042)

Madame, Monsieur,

Dans le cadre de la prise en charge de votre COVID-19, nous vous proposons de participer à une recherche interventionnelle nommée « POLYCOR».

**INTRODUCTION**

Cette recherche a pour but d’évaluer la sécurité et l’efficacité du XAV-19 dans le traitement de votre maladie.

Le CHU de Nantes est le promoteur de cette recherche, c’est à dire qu’il en est responsable et qu’il l’organise.

Votre participation à la recherche, au cas où vous donneriez votre accord, ne pourra vous être confirmée qu’à la condition que vous remplissiez tous les critères d’inclusion pour participer à cette recherche.

- Le médecin-investigateur, c’est-à-dire le médecin hospitalier qui vous suit dans le cadre de cette étude, vous a donné des explications. Elles sont résumées dans ce document intitulé « note d’information ». Nous vous invitons à le lire attentivement avant de vous décider. Vous disposez d’un délai de réflexion de quelques heures selon l’urgence de votre prise en charge avant de prendre votre décision et vous avez la possibilité d’en discuter avec votre médecin traitant ou vos proches. Si après lecture de cette note d’information vous ne souhaitez pas participer à cette étude, vous n’aurez pas à vous justifier et bénéficierez de la même qualité de soins.
- Si vous acceptez de participer à cette recherche, nous vous demanderons de dater et signer une attestation de consentement. Cette attestation sera aussi signée par l’investigateur. Cette signature confirmera que vous êtes d’accord pour participer à la recherche. Votre signature est indispensable, ainsi que celle de l’investigateur. Même après avoir signé pour donner votre accord de participation, vous garderez le droit d’interrompre à tout moment votre participation sans avoir à vous justifier.

# INFORMATION SUR LE PRODUIT A L’ETUDE

Le nouveau médicament testé dans l’étude s’appelle le XAV-19. Ce médicament de biotechnologie, fourni par le laboratoire français Xenothera, est un anticorps capable de bloquer l’entrée du virus SARS-CoV-2 dans les cellules humaines.

Cet anticorps est dit « glyco-humanisé », c’est-à-dire modifié pour être bien toléré. Il est d’origine porcine car ces animaux produisent des anticorps qui sont capables d’entraîner la neutralisation du virus sans risquer d’induire l’inflammation pouvant être associée aux traitements par d’autres types d’anticorps. La production de XAV-19 a suivi les hauts standards de sécurité requis pour l'injection à l'homme et les animaux impliqués dans sa production ont été élevés en suivant les règles éthiques et sanitaires requises pour la production d’un médicament.

Le XAV-19 est testé pour la première fois chez l’homme et n'a donc pas encore été approuvé par les Autorités de Santé pour une autorisation de mise sur le marché en France. Cependant, d’autres anticorps glyco-humanisés d’origine porcine issus de la même technologie ont déjà été utilisés chez l’homme, dans le cadre d’autres essais cliniques avec un profil de sécurité et tolérance très favorable.

# BUT DE LA RECHERCHE

La recherche est réalisée sous la responsabilité du Centre Hospitalier Universitaire de Nantes, en collaboration avec le CIMNA (Centre d'Immunomonitorage de Nantes Atlantique) et le CRTI (Centre de Recherche en Transplantation et Immunologie de Nantes).

Cette étude est divisée en deux phases (phase 2a et 2b). La phase 2a, phase à laquelle il vous est proposé de participer, inclura 16 patients qui recevront soit une des deux doses testées de XAV-19 (0,5mg/kg ou 2mg/kg) soit le placebo. Dans le premier groupe de 8 patients : 6 patients recevront le traitement à la dose de 0,5mg/kg et 2 patients le placebo. S’il n’y a pas de problème lié à la sécurité du traitement, les 8 autres patients seront inclus dans l’étude. Parmi ces 8 patients, 6 recevront le traitement à la dose de 2mg/kg et 2 patients le placebo. Cette étude a pour but d’évaluer la quantité de XAV-19 dans votre corps au fil du temps et de déterminer si le XAV-19, peut être administré en toute sécurité. Pour cela plusieurs examens et prises de sang seront réalisés tout au long de l’étude afin de collecter un certain nombre d’informations liées à l’administration du XAV-19. Cette phase a aussi pour objectif de déterminer la dose efficace pour traiter votre maladie. La dose choisie sera utilisée pour la phase 2b qui débutera à l’issue de la phase 2a. La phase 2b sera réalisée à plus grande échelle pour évaluer l’efficacité du traitement sur un plus grand nombre de patients.

Afin d’atteindre ces objectifs nous allons comparer chaque dose de traitement (0,5mg/kg et 2mg/kg), entre elles et avec le placebo.

Ce sera le tirage au sort fait au début de l’étude qui déterminera si vous recevrez le traitement ou le placebo. Cette étude est réalisée en double aveugle. Cela signifie que ni vous, ni l’équipe médicale ne saura dans quel groupe vous êtes. Ceci permettra à l’investigateur d’être plus objectif concernant les effets du produit à l’étude. Cette comparaison entre les 2 groupes de traitement se fera par l’évaluation des effets secondaires et de différents paramètres biologiques analysés au cours de l’étude.

Chaque phase se déroulera dans plusieurs centres hospitaliers français.

La durée de suivi de chaque patient dans l’étude est de 60 jours avec deux administrations du traitement XAV-19 (0,5mg/kg ou 2mg/kg) ou placebo: la première fois au 1^er^ jour (J1) et la seconde fois au 5^ème^ Jour (J5). Le traitement ou le placebo est administré par voie intraveineuse et la perfusion durera 30 à 60 minutes à chaque fois.

Dans le cadre de cette recherche, nous utiliserons les données médicales collectées au cours de votre prise en charge. Des échantillons de sang et d’urine seront prélevés pour le suivi de votre système immunitaire et le suivi de la molécule dans votre organisme. Dans le cadre de cette étude environ 250mL de sang seront prélevés pour la participation à cette étude.

# DEROULEMENT DE LA RECHERCHE

**Votre participation éventuelle à cette recherche durera 60 jours.**

Votre accord pour participer à cette recherche vous engage pendant toute la durée de la recherche à vous rendre à toutes les visites prévues dans le protocole, à savoir la visite de sélection, la visite d’inclusion, les visites de suivi, et la visite de fin de la recherche. Vous trouverez le calendrier de l’étude à la fin de ce document.

Le médecin-investigateur vous donnera également plus de détails au sujet de ces analyses et examens ainsi que la date à laquelle ils auront lieu.

- La visite de sélection : le médecin investigateur vérifiera les critères d’inclusion et vous proposera de participer à cette étude. Si vous acceptez de participer en signant le formulaire de consentement, le médecin-investigateur vous interrogera sur votre santé, vos antécédents médicaux et sur les médicaments que vous avez pris.

En outre, le médecin-investigateur effectuera les tests et examens suivants :

- Il vous fera passer un examen médical comprenant la mesure de votre taille, poids, tension artérielle, fréquence cardiaque et d'autres paramètres caractéristiques d'un examen clinique ;
- Il évaluera toutes les affections médicales que vous avez actuellement ou que vous avez eues auparavant et vous serez interrogé(e) sur les médicaments que vous prenez actuellement ou que vous avez pris dans le passé ;
- Il effectuera d'autres examens, notamment un prélèvement nasopharyngé (s’il n’a pas été déjà réalisé dans les 10 jours précédant l’inclusion), des analyses de sang et d'urine ;
- Si vous êtes une femme en âge d’avoir des enfants, un test de grossesse urinaire sera réalisé pour vérifier que vous n'êtes pas enceinte.
- Les virus suivants seront recherchés dans le sang (sérologie): VIH (virus de l’immunodéficience humaine, VHB (virus de l’hépatite B) et VHC (virus de l’hépatite C) ;

Le médecin-investigateur examinera les résultats des examens de sélection et vous informera si votre état de santé vous permet de participer à l’étude; dans le cas contraire, votre participation prendra fin après la période de sélection.

- La visite d’inclusion : Cette visite peut avoir lieu le même jour que la visite de sélection.

Une fois votre inclusion confirmée, le tirage au sort sera effectué pour déterminer votre groupe d’attribution.

- - Le médecin-investigateur fera un examen clinique et vérifiera qu’il n’y a pas eu de changement par rapport au premier examen réalisé lors de la visite de sélection ;
  - Il effectuera un prélèvement nasopharyngé (sauf si celui pour la visite de sélection a été réalisé dans le même établissement), un électrocardiogramme, des analyses de sang et d'urine ;
  - Vous recevrez alors votre première administration du traitement ou du placebo ;
  - Des analyses spécifiques seront réalisées avant et après l’administration du traitement. Ces analyses seront effectuées tout au long de l’étude pour le suivi de votre système immunitaire ainsi que pour le suivi de la molécule dans votre organisme ;
- Les visites de suivi : elles sont au nombre de 11 et seront effectuées en hospitalisation ou en consultation. Les examens pratiqués sont décrits dans le calendrier des visites.
- La visite de fin de la recherche : elle aura lieu au J29 ou à tout moment si vous sortez de l’étude prématurément. Les examens pratiqués sont décrits dans le calendrier des visites à la fin de cette note d’information.
- Un appel téléphonique sera fait au J60 pour connaître l’évolution de votre statut clinique.

# RISQUES DE LA RECHERCHE

Le fait de participer ou non à cette recherche ne changera rien au fait que vous devrez de toute façon recevoir un traitement pour le traitement de votre maladie COVID-19. Tout le reste de la prise en charge restera identique.

Cette recherche présente des risques et contraintes minimes par rapport à la prise en charge normale qui vous serait proposée si vous ne participiez pas à la recherche.

Les risques sont les effets indésirables possibles liés au traitement à l'étude et aux examens médicaux réalisés au cours de cette étude.

Le médecin-investigateur n'est pas en mesure de savoir quels patients auront ou non des effets indésirables. Certains effets indésirables disparaissent rapidement, certains peuvent être présents un certain temps ou certains peuvent même ne jamais disparaître.

- Quels sont les risques liés au traitement à l’étude ?

Les effets indésirables attendus chez l’homme avec le XAV-19 sont les effets décrits avec la plupart des traitements anticorps. Le XAV-19 pourrait provoquer des éruptions cutanées, douleur au point de la perfusion (thrombose), démangeaisons, difficultés à respirer, douleurs articulaires, maux de tête, de la fièvre, frissons. Des réactions allergiques plus sévères à type de gonflement du visage, de la langue, des sensations de malaise sont possibles. Cependant la conception spécifique du XAV-19 devrait permettre de réduire considérablement leur apparition.

- Quels sont les risques liés aux examens réalisés durant l’étude ?

Les risques associés aux prélèvements sanguins par voie intraveineuse : Comme pour toute prise de sang, il existe un risque d’hématome (un "bleu"), de douleur ou d’infection au site de prélèvement (à l’endroit où a été effectuée la piqûre).

- Quels sont les contraintes liées aux examens réalisés durant l’étude ?

Les visites de l’étude font partie intégrante du suivi pour les patients atteints de COVID-19. En cas de sortie d’hospitalisation après le J6, vous devrez revenir à l'hôpital pour les visites de suivi à J8, J15 et J29. Le transport sera pris en charge par l'étude pour ces visites.

En cas de problème, et à tout moment dans la recherche, vous devrez contacter l’investigateur ou le service de l’investigateur au _*_________________*

# BENEFICES ATTENDUS DE LA RECHERCHE

Si vous acceptez de participer à cette étude, nous ne pouvons pas vous assurer que vous en retirerez personnellement un bénéfice thérapeutique. Cependant, les examens supplémentaires et l’amélioration du suivi dans le cadre d’une étude clinique peuvent être considérés comme un bénéfice personnel.

Toutefois, notre hypothèse est que l’utilisation du XAV-19 pourrait permettre de réduire significativement la gravité et réduire la durée de votre maladie.

Les informations qui seront obtenues grâce à cette étude pourront contribuer à une meilleure connaissance de l’utilisation ou au développement de ce traitement pour soigner des malades atteints de la même pathologie que vous.

# ALTERNATIVES A LA RECHERCHE

Les traitements standards aujourd’hui utilisés au cas par cas comprennent des antiviraux, des antibiotiques et des traitements immuno-modulateurs (corticoides, anti-IL6, anti-IL1). Ces traitements restent compatibles avec la participation à cette étude.

Le médecin-investigateur pourra vous donner davantage d’informations sur les risques et bénéfices liés à votre participation à cette étude, comparé aux risques et bénéfices liés au schéma thérapeutique standard.

N’hésitez pas à lui poser la question avant de prendre votre décision et également tout au long de l’étude.

# FIN DE LA RECHERCHE

La recherche peut être interrompue à tout moment:

- par les autorités de santé,
- du fait du CHU de Nantes, promoteur de l’étude : si un élément nouveau survient, l’investigateur en sera informé et il vous transmettra alors les éléments susceptibles de modifier votre participation.
- du fait de l’investigateur, pour des raisons médicales vous concernant : si c’est une étude médicament, il peut décider à tout moment d’arrêter l’administration du produit à l’étude (par exemple à cause d’un effet secondaire ou d’une évolution de votre état de santé) et vous en informera.
- Si vous décidez de participer à cette recherche, il s’agira d’un acte volontaire. Vous pourrez à tout moment décider d’arrêter votre participation, sans pénalité ni préjudice. Dans ce cas, vous devrez informer l’investigateur de votre décision.

En cas d’arrêt prématuré de la recherche ou en fin de recherche il vous sera proposer un suivi médical spécialisé, par un infectiologue à un mois de votre sortie, puis par un pneumologue à 3 mois de votre pneumopathie pour surveiller l’évolution de vos symptômes après la sortie de votre hospitalisation.

Dans tous les cas, la qualité de votre prise en charge ne sera pas diminuée.

# FRAIS SUPPLEMENTAIRES

Votre participation à cette recherche n'engendrera pour vous aucun frais supplémentaire par rapport à ceux que vous auriez dans la prise en charge habituelle de cette maladie. Si vous sortez de l’hôpital avant la fin de l’étude et que vous devez revenir à l'hôpital pour une visite en ambulatoire ou pour un prélèvement sanguin spécifique de cette étude, le transport sera pris en charge par l'étude.

# VOS DROITS PENDANT LA RECHERCHE

- **Secret professionnel**

Le personnel impliqué dans la recherche est soumis au secret professionnel, tout comme votre médecin traitant.

Sauf avis contraire de votre part, votre médecin traitant pourra être informé de votre participation.

- **Accès aux données vous concernant - Traitement des données - confidentialité**

Dans le cadre de cette recherche, un traitement informatique de vos données personnelles va être mis en œuvre : cela permettra d’analyser les résultats de la recherche et de remplir l’objectif de la recherche.

Pour cela, les données médicales vous concernant seront recueillies dans un cahier électronique (eCRF) auquel aura accès le Promoteur de la recherche (CHU de Nantes).

Afin d’assurer leur confidentialité, ces données seront identifiées par un numéro de code et vos initiales.

Vos données pourront, dans des conditions assurant leur confidentialité via des plateformes ou serveurs sécurisés, faire l’objet de transferts nationaux ou internationaux (par exemple aux personnes ou sociétés agissant pour le compte du CHU ou aux autorités sanitaires habilitées) pour cette étude, ou pour d’autres recherches ultérieures, exclusivement à des fins scientifiques.

Vos données seront susceptibles d’être exploitées dans le cadre de publications ou de communications. Dans ce cas, votre anonymat sera préservé.

Conformément aux dispositions de la loi relative à l’informatique aux fichiers et aux libertés (loi modifiée du 6 janvier 1978), de la loi n° 2018-493 du 20 juin 2018 relative à la protection des données personnelles et du Règlement (UE) 2016/679 du Parlement européen et du Conseil du 27 avril 2016 relatif à la protection des personnes physiques à l'égard du traitement des données à caractère personnel et à la libre circulation de ces données (RGPD), vous disposez d’un droit d’accès, de rectification, de portabilité, de limitation et d’opposition du traitement de vos données personnelles. Quant au droit d’effacement, si vous décidez de retirer votre consentement pour participer à la recherche, les données obtenues avant que celui-ci n'ait été retiré seront utilisées conformément à l’article L.1122-1-1 du CSP. Les données recueillies après le retrait de votre consentement ne seront pas utilisées pour cette recherche et resteront destinées à l’usage strict du soin. Vous pouvez également porter une réclamation auprès d'une autorité de contrôle (CNIL pour la France : <https://www.cnil.fr/fr/webform/adresser-une-plainte/>).

Vous pouvez retirer votre consentement à cette utilisation ultérieure ou exercer votre faculté d'opposition à tout moment.

Vous pouvez également accéder directement ou par l’intermédiaire d’un médecin de votre choix à l’ensemble de vos données médicales en application des dispositions de l’article L 1111-7 du Code de la Santé Publique.

Vos données seront conservées tout au long de la recherche. Après la fin de la recherche, elles seront archivées pour une durée conforme aux dispositions réglementaires, puis détruites.

**Pour en savoir plus ou exercer vos droits concernant vos données, voir en fin de document, paragraphe « vos contacts ».**

- **Accès aux résultats globaux de la recherche**

A la fin de la recherche, et à votre demande, vous pourrez être informé(e) par l’investigateur des résultats globaux de cette recherche (dès qu’ils seront disponibles).

# VOS OBLIGATIONS PENDANT LA RECHERCHE

- **Vos obligations**

Vous devrez informer l’investigateur de tous les médicaments que vous prenez.

Vous devrez aussi l’informer immédiatement de tout évènement ou effet indésirable éventuellement rencontré au cours de votre participation à la recherche.

Vous devrez enfin vous rendre aux visites prévues.

- **Protection sociale**

Pour pouvoir participer à cette recherche vous devez être affilié(e) ou bénéficier d’un régime de sécurité sociale.

- **Modalités de participation à une autre recherche**

Pendant toute la durée de l’étude et jusqu’à trois mois après la fin de celle-ci, il ne vous sera pas possible de participer à une autre étude clinique avec un médicament à l’essai.

# LE CADRE REGLEMENTAIRE

**Cette recherche est conforme :**

- Aux articles L. 1121-1 à L. 1126-12 du code de la santé publique relatifs aux recherches impliquant la personne humaine

- A la loi « Informatique et Libertés » du 6 janvier 1978 modifiée et la loi n° 2018-493 du 20 juin 2018 relative à la protection des données personnelles

- au Règlement (UE) 2016/679 du Parlement européen et du Conseil du 27 avril 2016 relatif à la protection des personnes physiques à l'égard du traitement des données à caractère personnel et à la libre circulation de ces données (RGPD)

Vous pouvez retrouver tous ces textes sur le site [http://www.legifrance.gouv.fr](http://www.legifrance.gouv.fr/)

**Conformément aux dispositions réglementaires :**

- Le CHU de Nantes organise cette recherche en tant que « promoteur ». Il a souscrit un contrat d’assurance garantissant sa responsabilité civile et celle de tout intervenant auprès de la compagnie HDI GLOBAL SE (Contrat n° 0100775930012 200010).
- Cette recherche a reçu l’avis favorable du Comité de Protection des Personnes XXXX le …../……/………... La recherche a aussi reçu l’autorisation de l’ANSM (Agence Nationale de Sécurité du Médicaments et des Produits de Santé), le …../……/………..
- Le centre a reçu une Autorisation de lieux de recherche.

# DEVENIR DE VOS ECHANTILLONS PRELEVES AU COURS DE LA RECHERCHE

Si vous êtes d’accord, les échantillons biologiques résultant de votre prise en charge seront conservés dans une biocollection à la fin de la recherche. Cette biocollection a pour but d’identifier de possibles biomarqueurs liés à la gravité de la maladie et liés à la réponse au traitement. Vos échantillons pourront être utilisés pour d’autres recherches sur ce thème. Un formulaire de consentement dit de biocollection (différent de celui portant sur la recherche expliquée dans la présente note) va vous être soumis. L’investigateur ou un membre de son équipe vous fournira toutes les explications nécessaires et répondra à toutes vos questions. Si vous acceptez que vos échantillons soient conservés dans cette biocollection, vous devrez signer le formulaire.

# VOS CONTACTS :

Pour toute question concernant l’étude, retrait de consentement, ou pour exercer vos droits concernant vos données (accès, rectification, etc…), votre contact privilégié est :

**L’investigateur coordonnateur de la recherche :**

Dr GABORIT Benjamin

🖂 Département des maladies infectieuses, CHU de Nantes 44093 Nantes Cedex 1 - France

🕿 02 44 76 82 92

Pour toute question générale sur le traitement de vos données, votre contact privilégié est :

**Le promoteur de la recherche, responsable du traitement :**

CHU de Nantes, direction de la recherche

5 allée de l’Ile Gloriette, 44093 NANTES Cedex 1

**Le Délégué à la Protection des Données (DPO) :**

[vosdonneespersonnelles@chu-nantes.fr](mailto:vosdonneespersonnelles@chu-nantes.fr)

# EN RESUME…

Votre participation à cette recherche est libre. Vous pouvez refuser d’y participer.

De plus, vous pouvez à tout moment retirer votre consentement, sans préjudice.

Si vous décidez de refuser de participer à la recherche ou si vous décidez d’arrêter votre participation pendant la recherche :

- cela n’aura aucune conséquence sur la qualité des soins qui vous seront donnés
- vous devez simplement en informer l’investigateur.

Lorsque vous aurez lu cette note d’information et obtenu les réponses aux questions que vous vous posez en interrogeant l’investigateur (Dr/Pr…………………), il vous sera demandé, si vous en êtes d’accord, de donner votre consentement écrit en signant le formulaire préparé à cet effet.

Votre participation à la recherche, au cas où vous donneriez votre accord, ne pourra vous être confirmée qu’à la condition que vous remplissiez tous les critères d’inclusion.

Vous pouvez prendre votre temps avant de nous donner votre réponse.

Au cours de ce délai de réflexion, vous pouvez bien entendu continuer par téléphone à poser toutes les questions que vous souhaitez à l’investigateur (Dr/Pr.…………………….. tél………………………..).

Nous vous prions d’agréer, Madame, Monsieur, l’expression de nos sentiments les plus respectueux.

Dr Benjamin Gaborit, médecin coordonnateur et investigateur principal,

et toute l’équipe médicale en charge de cette recherche

*Vous devez conserver un exemplaire de ce document.*

**ANNEXES**

# ANNEXE 1 : CALENDRIER DES VISITES DE LA RECHERCHE

| **Examens** | 0 à 2 jours avant l’inclusion | J1  (visite d’inclusion) | J2 | J3 | J4 | J5 | J6 | J7** | J8 | J11** | J13** | J15 | J29 | J60** | Arrêt prématuré |
| --- | --- | --- | --- | --- | --- | --- | --- | --- | --- | --- | --- | --- | --- | --- | --- |
| Information du patient | X |  |  |  |  |  |  |  |  |  |  |  |  |  |  |
| Signature du consentement | X |  |  |  |  |  |  |  |  |  |  |  |  |  |  |
| Tirage au sort |  | X |  |  |  |  |  |  |  |  |  |  |  |  |  |
| Antécédents médicaux | X |  |  |  |  |  |  |  |  |  |  |  |  |  |  |
| Examen Clinique et signes vitaux | X | X | X | X | X | X | X | X | X | X | X | X | X | X | X |
| Administration XAV-19 ou placebo |  | X |  |  |  | X |  |  |  |  |  |  |  |  |  |
| Prélèvement nasopharyngé | * | X |  |  |  |  |  |  | X |  |  | X | X |  | X |
| Electrocardiogramme |  | X |  |  |  |  |  |  |  |  |  |  |  |  |  |
| Sérologie | X |  |  |  |  |  |  |  |  |  |  |  |  |  |  |
| Test de grossesse urinaire | X |  |  |  |  |  |  |  |  |  |  |  |  |  |  |
| Analyses sanguine et bandelette urinaire | X | X |  | X |  | X |  |  | X |  |  | X | X |  | X |
| Prélèvements sanguins pour la recherche |  | X |  | X |  | X |  |  | X |  |  | X | X |  |  |
| Traitements concomitants | X | X | X | X | X | X | X | X | X | X | X | X | X | X | X |
| Evènements indésirables | X | X | X | X | X | X | X | X | X | X | X | X | X | X | X |

**si non réalisé dans les 10 jours avant l’inclusion*

*** seront des appels téléphoniques si vous êtes sortis d’hospitalisation*

# ANNEXE 2 : CRITERES D’INCLUSION ET D’EXCLUSION DE L’ETUDE

Critères d'inclusion :

1. Volonté et capacité de donner un consentement éclairé écrit avant de démarrer les procédures de l’étude

2. Homme ou femme ≥ 18 ans et ≤ 85 ans

3. Hospitalisation pour COVID-19

4. RT-PCR positive pour le SRAS-CoV-2 dans tout échantillon corporel (nasopharynx, salive, crachats) ≤ 10 jours avant l'inclusion

5. Preuve d'une atteinte pulmonaire (à l'examen des poumons [râles/crépitements]) et/ou d'une imagerie de la poitrine (radiographie ou tomographie informatisée)

6. Nécessitant une supplémentation en O2 ≤ 6L/min à la visite de sélection

7. Nécessitant une supplémentation en O2 avec SpO2 ≥ 94% sous oxygénothérapie à la visite de sélection

8. Première apparition des symptômes du COVID-19 ≤ 10 jours, avec fièvre et/ou frissons, maux de tête, myalgies, toux, essoufflement, selon ce qui est apparu en premier

9. Les femmes en âge de procréer doivent avoir un test de grossesse urinaire négatif le jour de l'inclusion

10. Tous les sujets masculins sexuellement actifs doivent accepter d'utiliser une méthode de contraception adéquate pendant toute la durée de l'étude et pendant 90 jours après la dernière dose du médicament à l'étude et accepter de ne pas faire de don de sperme jusqu'à la fin de l'étude, ou pendant 90 jours après la dernière dose de XAV-19, selon la période la plus longue

11. Patients bénéficiant de la sécurité sociale française

Critères d'exclusion :

- 1. Preuve d’une défaillance de plusieurs organes (COVID-19 grave)
  2. Ventilation mécanique (y compris l'ECMO)
  3. Perfusion d'immunoglobulines ou de tout produit sanguin au cours des 30 derniers jours
  4. Maladie psychiatrique ou cognitive ou consommation de drogues ou d'alcool à des fins récréatives qui, de l'avis de l'enquêteur, affecterait la sécurité et/ou la conformité du sujet
  5. Maladie rénale en phase terminale (DFGe < 15 ml/min/1,73 m2)
  6. Cirrhose du foie au stade C selon l’échelle Child-Pugh
  7. Insuffisance cardiaque décompensée
  8. **Patients avec antécédents de toxicomanie active**
  9. Allergie, hypersensibilité ou intolérance connue au médicament étudié ou à l'un de ses composants
  10. Les femmes en âge de procréer sans méthode contraceptive, ou dont le test de grossesse est positif, qui allaitent ou qui prévoient de devenir enceintes pendant la période d'étude
  11. Infection bactérienne actuelle documentée et non contrôlée.
  12. Réactions allergiques antérieures graves (grade 3) à une transfusion de plasma
  13. Patient participant à un autre essai clinique interventionnel
  14. Espérance de vie estimée à moins de 6 mois
  15. Patient sous tutelle ou curatelle

Je soussigné(e)

M^e^, M. *(rayer la mention inutile*) (*prénom*, NOM) *………….….….…………*………………………………………… …………….…………………………………………………………………………………………………………………………,

Date de naissance : …../……/……..

**accepte librement et volontairement de participer à la recherche** **référencée ci-dessus,** coordonnée par le Docteur Benjamin Gaborit et organisée par le CHU de Nantes, promoteur de la recherche.

**Etant entendu que :**

- L’investigateur qui m’a informé(e) et a répondu clairement à toutes mes questions, m’a précisé que ma participation est libre et que je peux me retirer de la recherche à tout moment.
- J’atteste ne pas faire l'objet de mesure de protection (tutelle, curatelle, sauvegarde de justice), en outre je confirme être affilié(e) ou bénéficier d'un régime de sécurité sociale.
- Il m'a été préalablement remis une note d'information sur cette recherche précisant son but, sa méthodologie, ses bénéfices attendus et ses risques prévisibles.
- Je pourrai avoir communication par l’investigateur, au cours ou à l’issue de la recherche, des informations qu’il détient concernant ma santé.
- Je suis parfaitement conscient(e) que je peux retirer à tout moment mon consentement à ma participation à cette recherche et cela quelles que soient mes raisons et sans supporter aucune responsabilité, mais je m’engage dans ce cas à en informer l’investigateur. Le fait de ne plus participer à cette recherche ne portera pas atteinte à mes relations avec cet investigateur, ni à la qualité des soins qui me seront donnés.
- J’accepte que mon médecin traitant soit informé de ma participation à la recherche :

 Oui, j’accepte  Non, je refuse

- Je pourrai à tout moment demander des informations complémentaires à l’investigateur.
- Si je le souhaite, à son terme, je serai informé(e) par l’investigateur des résultats globaux de cette recherche.
- Mon consentement ne décharge en rien l’investigateur et le promoteur de l’ensemble de leurs responsabilités et je conserve tous mes droits garantis par la loi.
- Je ne pourrai pas participer à une autre recherche interventionnelle pendant toute ma participation à cette recherche.
- J’accepte que les données enregistrées à l’occasion de cette recherche puissent faire l’objet d’un traitement informatisé par le promoteur ou pour son compte, et j’atteste avoir été informé de tous mes droits concernant mes données personnelles selon les modalités décrites dans la note d'information en vigueur, qui m'a été transmise pour ce protocole.

| **PERSONNE SE PRETANT A LA RECHERCHE** | | |
| --- | --- | --- |
| **Date :**  **...... / ..... / ………..** | **Signature :** | |
| *Le cas échéant* : Attestation du consentement en cas d’impossibilité d’expression écrite de la personne qui se prête à la recherche par la personne de confiance, un membre de la famille ou un des proches | | |
| Date :  ...... / ..... / ……….. | Prénom NOM :  Lien avec la personne de prêtant à la recherche : | Signature : |

| **INVESTIGATEUR : J’atteste avoir pleinement expliqué à la personne signataire le but, les modalités ainsi que les risques potentiels de la recherche** | | |
| --- | --- | --- |
| **Date :**  **...... / ..... / ………..** | **NOM :** | **Signature :** |

Ce document est à réaliser en 2 exemplaires originaux : le premier doit être conservé par l’investigateur et le deuxième est remis à la personne donnant son consentement. En cas de duplicata, l’original est conservé par l’investigateur et une copie est remise à la personne ayant donné son consentement. En cas de triplicata, le promoteur récupèrera un des duplicata des consentements dans des enveloppes scellées tout au long de l’étude.
